# *LRRK2 *in Parkinson's disease – drawing the curtain of penetrance: a commentary

**DOI:** 10.1186/1741-7015-6-33

**Published:** 2008-11-05

**Authors:** Rejko Krüger

**Affiliations:** 1 Laboratory of Functional Neurogenomics, Center of Neurology and Hertie-Institute for Clinical Brain Research, University of Tübingen, Germany

## Abstract

Parkinson's disease is the most common neurodegenerative movement disorder and affects about 2% of the population over the age of 60 years. In 2004, mutations in the *LRRK2 *gene were first described and turned out to be the most frequent genetic cause of familial and sporadic Parkinson's disease and may account for up to 40% of patients in distinct populations. Based on these findings, Latourelle and colleagues show that the penetrance of the most common *LRRK2 *mutation is higher in patients with familial compared with sporadic Parkinson's disease and identified a substantial number of affected relatives of mutation carriers not presenting with a *LRRK2 *mutation themselves. This commentary discusses the role of genetic and/or environmental susceptibility factors modulating the expressivity of the disease trait, how these factors may contribute to the phenomenon of phenocopies in genetically defined Parkinson's disease pedigrees, and how the findings of Latourelle and colleagues, published this month in *BMC Medicine*, relate to current concepts of genetic counselling.

## 

Parkinson's disease (PD) is the most common neurodegenerative movement disorder, and has been regarded as a prototypic non-genetic disorder for a long time. First insight into the genetic contribution to the typical Parkinsonian phenotype came from large families with classical Mendelian inheritance of the disease trait. The discovery of disease-causing mutations in the *alpha-synuclein *gene for autosomal dominant forms and in the *Parkin, PINK1 *and *DJ-1 *gene for autosomal recessive forms of PD allowed a first insight into the molecular mechanisms that mediate neurodegeneration in PD. Heterozygous mutations in the *alpha-synuclein *gene and homozygous mutations in the *Parkin, PINK1 *and *DJ-1 *gene show, in general, full penetrance and account for less than 1% of all PD cases [1]. Thus, due to the small proportion of PD patients that carried mutations in the respective genes, genetic testing and counselling of presymptomatic mutation carriers was not relevant and common recommendations limited molecular testing to symptomatic individuals. These premises have now changed due to the identification and characterization of mutations in the *LRRK2 *gene in PD [2,3].

Mutations in the *LRRK2 *gene encoding the leucine-rich, repeat kinase 2 protein are the most frequent genetic cause of PD known to date. Clinicogenetic studies clearly showed that the majority of all *LRRK2 *mutation carriers present with symptoms indistinguishable from idiopathic PD [4-7]. To date, more than 50 variants are known, but only about 10% have been proven pathogenic and account for approximately 2% of sporadic and 10% of familial PD cases [4,8,9]. Initially identified in large families with autosomal dominant inheritance of PD, it turned out that mutations in the *LRRK2 *gene were also found in patients with the sporadic form of the disease (without positive family history). Among these mutations, a glycine to serine exchange in position 2019 of the peptide sequence (G2019S) is the most frequent worldwide with frequencies ranging from 1% in European to 40% in North African sporadic PD patients [10]. Increasing evidence for incomplete penetrance of *LRRK2 *mutations presented a major challenge for diagnostic testing and genetic counselling and highlighted the unmet need of validated estimates of population frequency and expressivity of the disease trait.

Focusing on the most common exemplary G2019S mutation, this month in *BMC Medicine*, Latourelle and colleagues addressed the problem of ascertainment bias that contributed to a wide range of penetrance estimates in previous studies on *LRRK2 *with lifetime penetrance ranging from 35% to 100% [5,11,12]. Indeed recruitment of patients for genetic studies from specialized institutions and/or inclusion of volunteer patients are suspected to overestimate mutation penetrance due to over-representation of families with multiple affected individuals based on an increased awareness of the disease [13]. Latourelle and colleagues used an elegant statistical approach to correct for inflated penetrance in their family-based study by using affected sib-pairs as a selection criterion and assessed penetrance for the parents irrespective of their disease status. Sufficiently powered population-based studies are still not available and are difficult to perform due to economical and ethical issues. Therefore, the present study represents a first step to overcoming estimates based on studies that were critically exposed to selection bias towards multiplex families, leading to overestimation of penetrance.

Latourelle and colleagues confirmed a reduced penetrance of 67% of the G2019S mutation in their family-based sample. Interestingly, the age-dependent penetrance of the G2019S mutation at age 85 years was nearly doubled in familial PD compared with randomly ascertained sporadic PD patients. Based on the assumption that overestimation of penetrance in these families is unlikely owing to the given statistical approach, this provides strong support for the existence of additional genetic or environmental modifiers of penetrance in these families. The potential impact of these currently unknown modifiers for disease expressivity culminates in the observed number of more than 14% phenocopies (relatives with PD that do not share the *LRRK2 *mutation) in G2019S-positive families [11]. This illustrates that these susceptibility factors may confer risk irrespective of G2019S status and may suffice to cause the PD phenotype in a subset of family members.

Is there evidence for the presence of additional susceptibility factors in genetically defined PD families? Indeed the phenomenon of phenocopies has been described in many of the original multiplex families with established PD-associated gene mutations, that is, *alpha-synuclein *and *Parkin *[14,15]. These have been typically explained by the population-based PD prevalence of 200 cases per 100,000 population and regarded as 'independent' [15,16]. However, the observed role of heterozygous mutations in autosomal recessive genes for PD (*Parkin, PINK1*) as potential predisposing factors for PD and the substantial proportion of more than 15% phenocopies in a large pedigree with *Parkin*-related PD strongly support the idea of an intrinsic susceptibility profile aggregating in the respective family background [15,17,18].

What do we already know about implicated genetic susceptibility factors? As can be deduced from existing studies, the modifiers of penetrance in *LRRK2*-related PD are not related to gender or ethnicity [6,11,19]. Indeed, independent genetic modifiers have been identified by large association studies in sporadic PD and may also modify penetrance in familial PD. In this context, several studies on functional polymorphisms in the promoter and 3'-end region of the *alpha-synuclein *gene revealed increased alpha-synuclein levels associated with an increased risk of developing PD [20,21]. Thus, polymorphisms in genes identified in familial PD may exert a disease-modulating effect that, in concert with other genetic or environmental susceptibility factors, may drive the accumulated risk over a critical threshold to cause neurodegeneration.

This may indicate that the same genes that encode fully penetrant mutations may provide minor alleles that may contribute to the penetrance-modifying effect leading to familial clusters of PD (Figure 1). However, recent studies in patients with co-occurrence of the *LRRK2 *and *Parkin *mutations found no evidence that heterozygous mutations in the *Parkin *gene, that are suspected to predispose to PD, modulate the expressivity of *LRRK2*-related PD [22,23]. This underscores the need for additional genetic and epidemiological studies in the genetically defined subgroup of G2019S-related PD that may help to dissect hereditary modifiers of penetrance and environmental factors influencing disease expressivity – the latter approach being unexpectedly fruitless in the past [24].

**Figure 1:**
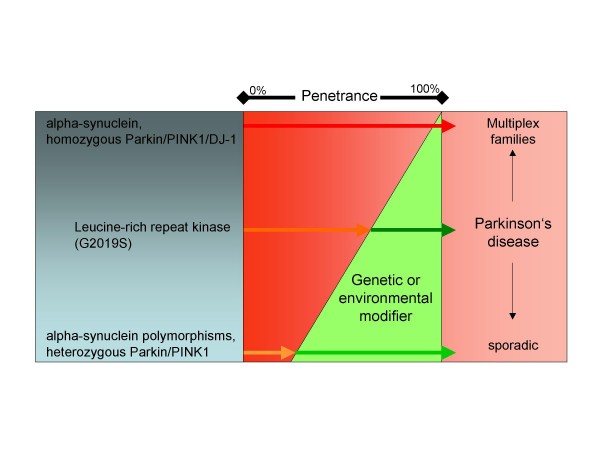
**Schematic view of the variable penetrance of the different known mutant genes and genetic risk factors involved in familial and sporadic Parkinson's disease illustrating current hypotheses on the impact of genetic modifiers in expressivity of the disease**. The higher the penetrance of a certain genetic trait, the more likely a familial clustering of the disease is observed, while lower penetrance is associated more with sporadic occurrence of Parkinson's disease. The G2019S mutation in the *LRRK2 *gene falls in the middle of this spectrum and may require the impact from additional susceptibility factors for full penetrance.

Until firm conclusions on the relative penetrance of identified disease genes like *LRRK2 *are available, the value of screening for mutations in asymptomatic family members of *LRRK2*-mutation carriers should be considered with caution, because until now no neuroprotective therapy has been implemented and symptomatic treatment is performed regardless of the presence or absence of mutations in known genes.

## Abbreviations

PD: Parkinson's disease

## Pre-publication history

The pre-publication history for this paper can be accessed here:



## References

[B1] Schiesling C, Kieper N, Seidel K, Krüger R (2008). Familial PD – genetics, clinical phenotype and neuropathology in relation to the common sporadic form of the disease. Neuropathol Appl Neurobiol.

[B2] Zimprich A, Biskup S, Leitner P, Lichtner P, Farrer M, Lincoln S, Kachergus J, Hulihan M, Uitti RJ, Calne DB, Stoessl AJ, Pfeiffer RF, Patenge N, Carbajal IC, Vieregge P, Asmus F, Muller-Myhsok B, Dickson DW, Meitinger T, Strom TM, Wszolek ZK, Gasser T (2004). Mutations in LRRK2 cause autosomal-dominant parkinsonism with pleomorphic pathology. Neuron.

[B3] Paisan-Ruiz C, Jain S, Evans EW, Gilks WP, Simon J, Brug M van der, Lopez de Munain A, Aparicio S, Gil AM, Khan N, Johnson J, Martinez JR, Nicholl D, Carrera IM, Pena AS, de Silva R, Lees A, Marti-Masso JF, Perez-Tur J, Wood NW, Singleton AB (2004). Cloning of the gene containing mutations that cause PARK8-linked Parkinson's disease. Neuron.

[B4] Berg D, Schweitzer K, Leitner P, Zimprich A, Lichtner P, Belcredi P, Brussel T, Schulte C, Maass S, Nagele T (2005). Type and frequency of mutations in the LRRK2 gene in familial and sporadic Parkinson's disease. Brain.

[B5] Lesage S, Durr A, Tazir M, Lohmann E, Leutenegger AL, Janin S, Pollak P, Brice A (2006). LRRK2 G2019S as a cause of Parkinson's disease in North African Arabs. N Engl J Med.

[B6] Healy DG, Falchi M, O'Sullivan SS, Bonifati V, Durr A, Bressman S, Brice A, Aasly J, Zabetian CP, Goldwurm S, Ferreira JJ, Tolosa E, Kay DM, Klein C, Williams DR, Marras C, Lang AE, Wszolek ZK, Berciano J, Schapira AH, Lynch T, Bhatia KP, Gasser T, Lees AJ, Wood NW, International LRRK2 Consortium (2008). Phenotype, genotype, and worldwide genetic penetrance of LRRK2-associated Parkinson's disease: a case-control study. Lancet Neurol.

[B7] Hulihan MM, Ishihara-Paul L, Kachergus J, Warren L, Amouri R, Elango R, Prinjha RK, Upmanyu R, Kefi M, Zouari M, Sassi SB, Yahmed SB, El Euch Fayeche G, Matthews PM, Middleton LT, Gibson RA, Hentati F, Farrer MJ (2008). LRRK2 Gly2019Ser penetrance in Arab-Berber patients from Tunisia: a case-control genetic study. Lancet Neurol.

[B8] Di Fonzo A, Rohe CF, Ferreira J, Chien HF, Vacca L, Stocchi F, Guedes L, Fabrizio E, Manfredi M, Vanacore N, Goldwurm S, Breedveld G, Sampaio C, Meco G, Barbosa E, Oostra BA, Bonifati V (2005). A frequent LRRK2 gene mutation associated with autosomal dominant Parkinson's disease. Lancet.

[B9] Mata IF, Wedemeyer WJ, Farrer MJ, Taylor JP, Gallo KA (2006). LRRK2 in Parkinson's disease: protein domains and functional insights. Trends Neurosci.

[B10] Goldwurm S, Zini M, Mariani L, Tesei S, Miceli R, Sironi F, Clementi M, Bonifati V, Pezzoli G (2007). Evaluation of LRRK2 G2019S penetrance. Neurology.

[B11] Latourelle JC, Sun M, Lew MF, Suchowersky O, Klein C, Golbe LI, Mark MH, Growdon JH, Wooten F (2008). The G2019S mutation in LRRK2 is not fully penetrant: The GenePD study. BMC Medicine.

[B12] Ozelius LJ, Senthil G, Saunders-Pullman R, Ohmann E, Deligtisch A, Tagliati M, Hunt AL, Klein C, Henick B, Hailpern SM, Lipton RB, Soto-Valencia J, Risch N, Bressman SB (2006). LRRK2 G2019S as a cause of Parkinson's disease in Ashkenazi Jews. N Engl J Med.

[B13] Begg CB (2002). On the use of familial aggregation in population-based case probands for calculating penetrance. J Natl Cancer Inst.

[B14] Polymeropoulos MH, Lavedan C, Leroy E, Ide SE, Dehejia A, Dutra A, Pike B, Root H, Rubenstein J, Boyer R, Stenroos ES, Chandrasekharappa S, Athanassiadou A, Papapetropoulos T, Johnson WG, Lazzarini AM, Duvoisin RC, Di Iorio G, Golbe LI, Nussbaum RL (1997). Mutation in the alpha-synuclein gene identified in families with Parkinson's disease. Science.

[B15] Pramstaller PP, Schlossmacher MG, Jacques TS, Scaravilli F, Eskelson C, Pepivani I, Hedrich K, Adel S, Gonzales-McNeal M, Hilker R, Kramer PL, Klein C (2005). Lewy body Parkinson's disease in a large pedigree with 77 Parkin mutation carriers. Ann Neurol.

[B16] Schrag A, Ben-Shlomo Y, Quinn NP (2000). Cross-sectional prevalence survey of idiopathic Parkinson's disease and Parkinsonism in London. BMJ.

[B17] Hedrich K, Hagenah J, Djarmati A, Hiller A, Lohnau T, Lasek K, Grunewald A, Hilker R, Steinlechner S, Boston H, Kock N, Schneider-Gold C, Kress W, Siebner H, Binkofski F, Lencer R, Munchau A, Klein C (2006). Clinical spectrum of homozygous and heterozygous PINK1 mutations in a large German family with Parkinson disease: role of a single hit?. Arch Neurol.

[B18] Klein C, Lohmann-Hedrich K, Rogaeva E, Schlossmacher MG, Lang AE (2007). Deciphering the role of heterozygous mutations in genes associated with parkinsonism. Lancet Neurol.

[B19] Haugarvoll K, Rademakers R, Kachergus JM, Nuytemans K, Ross OA, Gibson JM, Tan EK, Gaig C, Tolosa E, Goldwurm S, Guidi M, Riboldazzi G, Brown L, Walter U, Benecke R, Berg D, Gasser T, Theuns J, Pals P, Cras P, Paul De Deyn P, Engelborghs S, Pickut B, Uitti RJ, Foroud T, Nichols WC, Hagenah J, Klein C, Samii A, Zabetian CP, Bonifati V, Van Broeckhoven C, Farrer MJ, Wszolek ZK (2008). Lrrk2 R1441C parkinsonism is clinically similar to sporadic Parkinson disease. Neurology.

[B20] Maraganore DM, de Andrade M, Elbaz A, Farrer MJ, Ioannidis JP, Kruger R, Rocca WA, Schneider NK, Lesnick TG, Lincoln SJ, Hulihan MM, Aasly JO, Ashizawa T, Chartier-Harlin MC, Checkoway H, Ferrarese C, Hadjigeorgiou G, Hattori N, Kawakami H, Lambert JC, Lynch T, Mellick GD, Papapetropoulos S, Parsian A, Quattrone A, Riess O, Tan EK, Van Broeckhoven C (2006). Collaborative analysis of alpha-synuclein gene promoter variability and Parkinson disease. JAMA.

[B21] Fuchs J, Tichopad A, Golub Y, Munz M, Schweitzer KJ, Wolf B, Berg D, Mueller JC, Gasser T (2008). Genetic variability in the SNCA gene influences alpha-synuclein levels in the blood and brain. FASEB J.

[B22] Dächsel JC, Mata IF, Ross OA, Taylor JP, Lincoln SJ, Hinkle KM, Huerta Ribacoba R, Blazquez M, Alvarez V, Farrer MJ (2006). Digenic parkinsonism: investigation of the synergistic effects of PRKN and LRRK2. Neurosci Lett.

[B23] Marras C, Klein C, Lang AE, Wakutani Y, Moreno D, Sato C, Yip E, Munhoz RP, Lohmann K, Djarmati A, Bi A, Rogaeva E (2008). LRRK2 and Parkin mutations in a family with parkinsonism-Lack of genotype-phenotype correlation. Neurobiol Aging.

[B24] Hardy J (2006). No definitive evidence for a role for the environment in the etiology of Parkinson's disease. Mov Disord.

